# XPO1-dependent nuclear export as a target for cancer therapy

**DOI:** 10.1186/s13045-020-00903-4

**Published:** 2020-06-01

**Authors:** Nancy G. Azizian, Yulin Li

**Affiliations:** 1 Center for Immunotherapy Research, Houston Methodist Research Institute, 6670 Bertner Avenue, Houston, TX 77030 USA; 2 Department of Medicine, Weill Cornell Medical College, New York, NY 10065 USA

**Keywords:** XPO1, CRM1, Nuclear export, Selective inhibitor of nuclear export (SINE), Selinexor, Cancer

## Abstract

Cellular homeostasis requires the proper nuclear-cytoplasmic partitioning of large molecules, which is often deregulated in cancer. XPO1 is an export receptor responsible for the nuclear-cytoplasmic transport of hundreds of proteins and multiple RNA species. XPO1 is frequently overexpressed and/or mutated in human cancers and functions as an oncogenic driver. Suppression of XPO1-mediated nuclear export, therefore, presents a unique therapeutic strategy. In this review, we summarize the physiological functions of XPO1 as well as the development of various XPO1 inhibitors and provide an update on the recent clinical trials of the SINE compounds. We also discuss potential future research directions on the molecular function of XPO1 and the clinical application of XPO1 inhibitors.

## Background

Eukaryotic cells have well-separated nuclear and cytoplasmic compartments. Proper cellular functions require the exchange of large molecules through the nuclear pore complex (NPC). Small molecules can passively diffuse through the NPC, whereas the transport of larger cargoes, including RNAs and proteins, requires various transport receptors of the importin beta superfamily [1, 2]. XPO1 is a major transport receptor, responsible for exporting proteins and multiple RNA species. Originally named as CRM1 (chromosomal region maintenance 1), XPO1 was identified in *Schizosaccharomyces pombe* as a gene required for maintaining higher-order chromosome structure [3]. Subsequently, it was shown to function as a shuttling protein, mediating the nuclear export of proteins and mRNAs in *Saccharomyces cerevisiae* and renamed as XPO1 (exportin 1) [4].

## Physiological functions of XPO1/CRM1

**Fig. 1:**
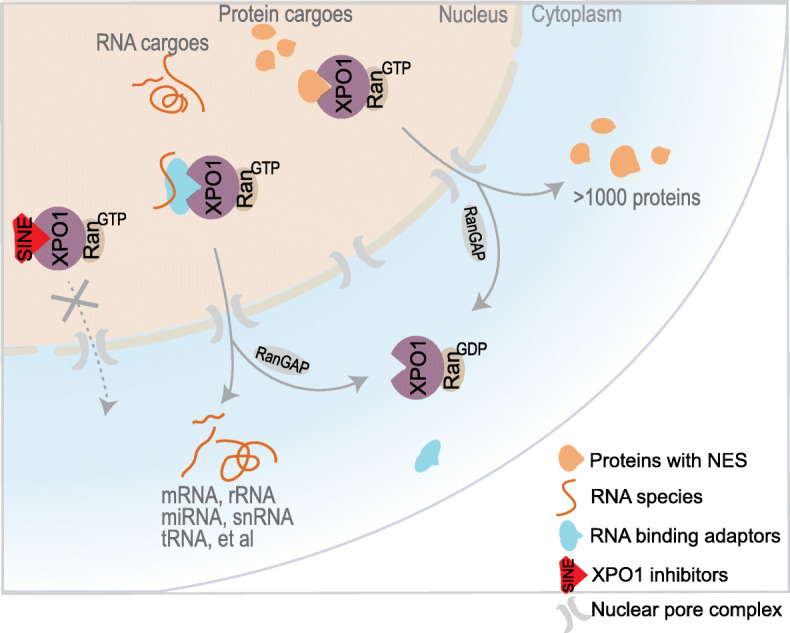
XPO1 mediates the nuclear export of hundreds of proteins and multiple RNA species

XPO1 is a nuclear export receptor with a pleiotropic role in transporting a plethora of proteins and RNA species, including rRNAs, snRNAs, mRNA, microRNAs, and tRNAs [5] (Fig. 1). XPO1 functions together with RAN GTPase, which provides the energy for transport and ensures the directionality of nuclear export [6]. In the nucleus, XPO1 binds to the nuclear export signal (NES) on its target proteins and to RAN in its active GTP-bound form (RAN-GTP). The complex is subsequently docked to NPC and passes through the nuclear membrane into the cytoplasm. Hydrolysis of RAN-GTP to RAN-GDP causes the disassembly of the complex and release of cargoes in the cytoplasm. The directionality of XPO1-mediated export is determined by the concentration gradient of RAN-GTP, which is predominantly confined to the nucleus [7] (Fig. 1). In addition to its role in nuclear-cytoplasmic transport during the interphase of cell cycle, XPO1/RAN regulates mitosis.

### Protein export

XPO1 is involved in the export of nearly 220 proteins bearing NESs [8]. Among these proteins, several tumor suppressors, including p53, BRCA1/2, and p27, have been extensively studied. Nuclear export blockade of tumor suppressor proteins has been postulated as the primary mechanism of action (MOA) for XPO1 inhibitors [9, 10]. However, many known oncoproteins, such as SNAIL, cyclins, TERT/telomerase, SURVIVIN, DNA topoisomerases, c-ABL, and YAP1, are also exported by XPO1 [8, 11]. The indiscriminate export of tumor suppressors and oncogenes by XPO1 argues against nuclear retention of tumor suppressors as the major MOA for XPO1 inhibitors. Indeed, XPO1 inhibitors have been demonstrated to exhibit antitumor activities independent of the function of key tumor suppressor proteins, including RB, p53, and p21 [12–14]. The number of proteins exported by XPO1 may have been remarkably underestimated by earlier studies. A recent deep proteomic characterization of XPO1 protein cargoes has identified > 700 export substrates from *S. cerevisiae*, > 1000 from *Xenopus* oocytes, and > 1050 from human cells. The protein partitioning data suggest broad XPO1 functions in the regulation of vesicle coat-assembly, centrosomes, autophagy, peroxisome biogenesis, cytoskeleton, ribosome maturation, translation, and mRNA degradation [15]. This study concludes that XPO1-mediated protein export is general and promiscuous and that the impaired export of tumor suppressors may be one of the multiple potential mechanisms of action for XPO1 inhibitors.

### RNA export

XPO1 has a major role in the nuclear export of multiple RNA species. First, XPO1 mediates the export of 40s and 60s ribonucleoprotein (RNP) complex in lieu of the naked ribosomal RNAs (rRNAs). Biogenesis of ribosomal subunits involves the synthesis of structural rRNAs and ribosomal proteins; their assembly into pre-ribosomal subunits in the nucleolus, export by XPO1; and further processing before gaining translational competency [16]. Second, XPO1 is critical for mRNA splicing by regulating the maturation of small nuclear RNAs (snRNAs). Following transcription in the nucleus, U snRNAs interact with the adaptor protein PHAX, RAN-GTP, and XPO1 to form an export-competent assembly. Exported U snRNAs are released in the cytoplasm, modified, and assembled into U snRNPs, before being shuttled back into the nucleus for further assembly into spliceosomes [17]. Third, XPO1 is involved in the export of other small non-coding RNAs, including microRNAs and tRNAs. microRNA and tRNA precursors are primarily exported by exportin 5 (XPO5) and exportin t (XPOT), respectively. However, XPO1 can mediate the alternative export of both microRNAs and tRNAs [18–22]. Fourth, XPO1 also exports mRNAs. mRNA is exported through either the bulk NXF1-mediated or the selective XPO1-mediated pathway [23, 24]. In particular, XPO1 and additional adaptor proteins with RNA binding properties, including LRPPRC, eIF4E, NXF3, and HuR, can preferentially export a subset of mRNAs encoding oncoproteins [25–28]. The diversity of the RNA species exported by XPO1 indicates that the inhibition of XPO1 may have a profound impact on different aspects of RNA metabolism.

### Export-independent function

XPO1/RAN complex carries out nuclear export function during interphase, with an intact nuclear membrane. Equally important, however, is the export-independent function of XPO1/RAN during mitosis [29, 30]. XPO1 was originally identified as CRM1, with an essential role in regulating mitosis and chromosomal structure. XPO1 has been shown to localize to mitotic kinetochores [31] and is required for microtubule nucleation [32]. It is also present at centrosomes during cell cycle and may be involved in the recruitment of centrosomal scaffold proteins and assembly of mitotic spindles [33]. The transport-independent function is not limited to XPO1/RAN, as other members of the importin beta family and nucleoporins also play a role in regulating spindle assembly and kinetochore function [29, 30].

## XPO1/CRM1 as an oncogenic driver and therapeutic target in cancer

XPO1 overexpression is a common feature among many human cancer types, including pancreatic, ovarian, glioma, lung, gastric, prostate, and colorectal cancers, and is associated with poor prognosis [34–40]. While amplified copy number may explain the XPO1 overexpression in some leukemia and lymphoma subtypes [41], for the majority of human cancers, the mechanism of XPO1 overexpression remains unknown. Genetic studies demonstrate a converse role for MYC and P53 in regulating XPO1 transcription [42]. The MYC overexpression and/or P53 loss of function observed in most human cancers are likely responsible for the widespread XPO1 overexpression. Interestingly, several XPO1 binding partners and/or adaptor proteins involved in nuclear export, namely RAN, HuR, eIF4E, LRPPRC, and NXF3, are also frequently overexpressed in human cancers and correlate with poor prognosis [24, 43–47], pointing to the aberrations of nuclear export machinery as a major hallmark of tumorigenesis. In addition to overexpression, XPO1 mutations have been found in 0.5–2.9% of solid and hematopoietic tumors, with the highest incidence in lymphomas [48]. A recent large-scale analysis of whole-exome and genome sequencing data from 42,793 patients has identified highly recurrent mutations of XPO1 (E571, R749, and D624) specifically in B cell malignancies. The E571K mutation is enriched in primary mediastinal B cell lymphoma (33%), classic Hodgkin lymphoma (14%), diffuse large B cell lymphoma (DLBCL) (2%), and chronic lymphocytic leukemia (CLL) (3%) [49]. Furthermore, XPO1^E571K^ mutation has been shown to cooperate with MYC and BCL2 in promoting lymphomagenesis. These observations suggest that gain-of-function mutations convert XPO1 to an oncogenic driver, particularly in B cell malignancies. The exclusive presence of XPO1 mutations in B cell malignancies is largely unknown but is likely related to somatic hypermutations of immunoglobulin genes, where the error-prone polymerase η may introduce a high frequency of A > T and C > G transversions [50–52]. Due to frequent deregulation in cancers, XPO1 has been identified as a therapeutic target in many tumor types. Recent CRISPR library and RNAi screens have validated XPO1 as a therapeutic target in sarcoma, DLBCL, multiple myeloma, and KRAS-mutant lung cancer [9, 53–55]. Specific XPO1 inhibitors have been extensively tested and demonstrated efficacy in a broad range of cancer types in preclinical studies.

## Development and preclinical study of XPO1/CRM1 inhibitors

The development of XPO1/CRM1 inhibitors dates back to early 1980s with the discovery of multiple classes of compounds and has been elegantly summarized in another review [56]. The first characterized XPO1 inhibitor, leptomycin B, was isolated from a strain of *Streptomyces* as an antifungal agent [57, 58]. Shortly following isolation and purification, its antitumor efficacy was examined in murine transplantation tumor models and demonstrated survival benefits [59]. However, a phase 1 trial of leptomycin B carried out in a small cohort of patients with various advanced cancers demonstrated marked malaise and anorexia, with no partial response [60]. Further clinical development of leptomycin B was therefore halted following the failed trial. XPO1/CRM1 was subsequently identified as the molecular target of leptomycin B with covalent binding at Cys528 [61]. Leptomycin B analogs, including ratjadones, anguinomycins, and KOS2464, also covalently bind to XPO1/CRM1 [62–64]. In particular, the semisynthetic KOS2464 has demonstrated significant therapeutic efficacy in several solid and hematopoietic tumor types with much reduced toxicity compared to leptomycin B [64].

In recent years, synthetic inhibitors of XPO1/CRM1, including PKF050-638, CBS9106, and selective inhibitors of nuclear export (SINE), have been developed. CBS9106 was shown to bind XPO1/CRM1, suppress its nuclear export activities, and induce XPO1/CRM1 protein degradation [65, 66]. Treatment with CBS9106 induces cell cycle arrest and apoptosis in a broad spectrum of cancer cells. Oral administration of CBS9106 significantly suppresses tumor growth and prolongs survival without a significant loss of body weight in a xenograft mouse model of multiple myeloma [65]. PKF050-638 originally identified as an inhibitor of HIV Rev protein also interferes with XPO1/CRM1 function [67]. Its N-azolylacrylate scaffold was later adopted by the SINE compounds, a series of structurally related small molecule inhibitors, including KPT-185, KPT-276, KPT-335, KPT-330 (selinexor), KPT-8602 (eltanexor), and SL-801 (felezonexor) [68, 69]. SINE compounds specifically bind to Cys528 in the cargo-binding groove of XPO1/CRM1. In contrast to leptomycin B, the covalent binding of SINE compounds to XPO1/CRM1 is slowly reversible, potentially explaining SINEs’ relatively low toxicity [63, 68]. The development of SINEs has enabled the translational application of nuclear export inhibitors as novel therapeutic agents. A number of SINE compounds have been tested extensively in preclinical settings, exhibiting efficacy in solid tumors [14, 70–74] and hematopoietic malignancies [75–79]. Among the investigated SINEs, KPT-330, KPT-8602, and SL-80 have been advanced to clinical trials. KPT-330 (selinexor) in particular has been evaluated in the majority of the trials and recently received FDA approval for resistant and relapsed multiple myeloma [80].

## Clinical trials of XPO1/CRM1 inhibitors

### Solid tumors

Single-agent selinexor has shown limited potential in clinical trials for the treatment of solid tumors. A phase 1 study of 189 patients with advanced solid tumors evaluated the safety and efficacy of selinexor (NCT01607905) [81]. Among the 157 evaluable patients, only one complete and six partial responses were observed (4%). Several phase 2 trials further tested single-agent selinexor for the treatment of specific solid tumors. A phase 2 study of selinexor in 56 patients with advanced de-differentiated liposarcoma did not find a significant difference in progression-free survival (PFS) (NCT02606461) [82]. In another phase 2 study of 14 patients with metastatic castration-resistant prostate cancer, single-agent treatment with selinexor led to > 50% reduction in PSA in only two patients. Of the eight patients with a measurable disease at baseline, two had a partial response and four had stable disease as their best radiographic response (NCT02215161). Notably, selinexor treatment was associated with significant toxicities, which limited future clinical application of selinexor in this patient population [83]. Single-agent selinexor was also tested in metastatic triple-negative breast cancer in a phase 2 trial (NCT02402764) with ten heavily pretreated patients. Although fairly well tolerated in these patients, selinexor treatment did not result in objective responses (OR) [84]. In this light, further clinical trials of selinexor and potentially other SINEs in solid tumors should focus on combination therapies. Indeed, several current clinical trials are evaluating combined application of selinexor and other drugs, such as ixazomib (a proteasome inhibitor) in advanced sarcoma (NCT03880123), paclitaxel and carboplatin in advanced ovarian and endometrial cancers (NCT02269293), gemcitabine and nab-paclitaxel in pancreatic cancer (NCT02178436), and several standard chemotherapy regimens for treatment of various advanced solid tumors (NCT02419495).

In contrast to the limited efficacy in solid tumors, selinexor as a single-agent or part of drug combinations has shown promising efficacy in clinical trials of hematopoietic cancers, including acute myeloid leukemia (AML), multiple myeloma, and non-Hodgkin lymphoma (NHL) (summarized in Table 1). Notably, selinexor in combination with several standard therapies has demonstrated superior efficacy in relapsed or refractory multiple myeloma, leading to a recent FDA approval of the selinexor/dexamethasone combination.

**Table 1:** Clinical trials of selinexor and eltanexor in hematological malignancies

Diseases	Drug(s)	Study phase	No. of patients	Outcome (ORR)	NCT number
RR AML	Selinexor	I	95	14%	NCT01607892
RR AML	Selinexor/MEC	I	21	39%	NCT02299518
AML	Selinexor/cytarabine/mitoxantrone	I	20	70%	NCT02573363
FLT3-mutated AML	Selinexor/sorafenib	IB/II	14	43%	NCT02530476
Pediatric RR leukemia	Selinexor/fludarabine/cytarabine	I	18	47%	NCT02212561
RR NHL	Selinexor	I	79	31%	NCT01607892
RR DLBCL	Selinexor	IIB	129	Ongoing	NCT02227251
RR DLBCL	Selinexor/RICE	I	23	Ongoing	NCT02471911
Advanced NHL	Selinexor/R-CHOP	IB/II	44	Ongoing	NCT03147885
RR CLL/NHL	Selinexor/ibrutinib	I	92	Ongoing	NCT02303392
DLBCL/AML	Selinexor/venetoclax	I	78	Ongoing	NCT03955783
RR MM	Selinexor/dexamethasone	I	59	50% vs 4%	NCT01607892
RR MM	Selinexor/dexamethasone	IIB	202	26%	NCT02336815
RR MM	Selinexor/dexamethasone/bortezomib	I/II	42	63%	NCT02343042
RR MM	Selinexor/dexamethasone/bortezomib	III	402	13.93 vs 9.46 months (PFS)	NCT03110562
RR MM/MDS	Eltanexor (KPT-8602)	I/II	119	Ongoing	NCT02649790

*Abbreviations*: *MEC* mitoxantrone, etoposide, and cytarabine; *RICE* rituximab, ifosfamide, carboplatin, and etoposide; *R-CHOP* rituximab, cyclophosphamide, doxorubicin, vincristine, and prednisone

### Acute myeloid leukemia

Selinexor has been tested in several clinical trials of AML. A phase 1 dose-escalation study examined the safety and efficacy of selinexor in 95 patients with relapsed or refractory (RR) AML (NCT01607892) [85]. Overall, 14% of the patients achieved an OR and 31% showed ≥ 50% decrease in bone marrow blasts. More commonly, however, selinexor has been tested in combination with other cytotoxic or targeted agents. A phase 1 study of selinexor combined with mitoxantrone, etoposide, and cytarabine in 21 patients with RR AML showed an overall response rate (ORR) of 39%, including 4 complete remissions (CR) and 2 complete remissions with incomplete hematologic recovery (CRi) (NCT02299518) [86]. Another phase 1 trial evaluated selinexor in combination with high-dose cytarabine and mitoxantrone for remission induction in 20 patients with either newly diagnosed or RR AML (NCT02573363) [87]. An ORR of 70% was observed, with 10 CR, 3 CRi, and 1 PR. In a phase 1b/2 clinical trial of FLT3-mutated refractory AML, selinexor was combined with sorafenib to target XPO1 and FLT3, respectively (NCT02530476). Selinexor/sorafenib combination induced CR or PR in six of 14 patients [88]. Selinexor was also combined with fludarabine and cytarabine for the treatment of pediatric RR acute leukemias and myelodysplastic syndrome (MDS) (NCT02212561). Seven of the 15 evaluable patients achieved CR or CRi, including five with no detectable minimal residual disease [89].

### Non-Hodgkin lymphoma

Based on promising preclinical findings, selinexor was also evaluated in clinical trials of NHLs. A phase 1 trial (NCT01607892) tested single-agent selinexor in 79 patients from various NHL subtypes, including DLBCL, Richter’s transformation, mantle cell lymphoma, follicular lymphoma, CLL, and double/triple-hit lymphomas [90]. Twenty-two (31%) of the 70 evaluable patients had an OR, including 4 CR and 18 PR, across all NHL subtypes. These findings suggest that single-agent selinexor has encouraging therapeutic efficacy in patients with RR NHL. A phase 2b study of single-agent selinexor is in progress in 129 patients with RR DLBCL (NCT02227251). In addition to single-agent use, selinexor is currently investigated in clinical trials for the treatment of advanced NHLs, in combination with other agents including RICE (rituximab, ifosfamide, carboplatin, and etoposide) (NCT02471911), R-CHOP (rituximab, cyclophosphamide, doxorubicin, vincristine, and prednisone) (NCT03147885), ibrutinib (NCT02303392), and venetoclax (NCT03955783).

### Multiple myeloma

Selinexor has been most extensively tested in multiple myeloma and recently received FDA approval. In an early phase 1 study, selinexor was tested either as a single-agent or in combination with dexamethasone in a cohort of heavily pretreated multiple myeloma patients (NCT01607892) [91]. Single-agent selinexor showed modest efficacy with an ORR of 4%. However, the drug combination was well tolerated with an ORR of 50% (1 CR and 5 PR in 12 patients). Thus, selinexor/dexamethasone combination is active in heavily pretreated multiple myeloma. The combination therapy was further evaluated in a phase 2b trial in a large cohort of heavily pretreated patient (NCT02336815) [92, 93]. These patients had previous exposure to bortezomib, carfilzomib, lenalidomide, pomalidomide, daratumumab, and an alkylating agent and had disease refractory to at least one proteasome inhibitor, one immunomodulatory agent, and daratumumab (triple-class refractory). A partial or better response was observed in 26% of the 122 patients, including two CR. Following the phase 2b trial, selinexor was approved by FDA in combination with dexamethasone, for the treatment of patients with RR multiple myeloma who had received at least four prior therapies and whose disease was refractory to at least two proteasome inhibitors, at least two immunomodulatory agents, and an anti-CD38 monoclonal antibody.

A large phase 1/2 trial of selinexor/dexamethasone combination plus other backbone treatments for MM is currently in progress for RR and newly diagnosed patients (NCT02343042). In this trial, the combination of selinexor/dexamethasone/bortezomib has led to an ORR of 63% in a cohort of 42 patients [94]. This preliminary finding has been further extended to a large phase 3 study including 402 RR patients (NCT03110562). Patients in the selinexor/bortezomib/dexamethasone arm had a median PFS of 13.93 months, compared to 9.46 months in patients in the bortezomib plus dexamethasone arm.

### Adverse effects in clinical trials

As XPO1 is widely expressed in normal tissues/cells and is required for mitosis, administration of XPO1 inhibitors is expected to cause systemic toxicities. Due to the reversible nature of the XPO1-SINE binding, selinexor is relatively well tolerated in most reported clinical trials. The most common non-hematological adverse events (AEs) are gastrointestinal disturbances, which are primarily grade 1 or 2 but can also be grade 3 [83, 93]. Additionally, asymptomatic hyponatremia and hypokalemia have also been commonly observed. The most common grade 3 or 4 AEs are hematologic, including thrombocytopenia, anemia, and neutropenia. Thrombocytopenia, in particular, is a distinct AE of selinexor due to the impaired thrombopoietin signaling and differentiation of stem cells into megakaryocytes [95]. One SINE compound, KPT-8602, has shown reduced general toxicity in preclinical studies due to limited penetration through the blood-brain barrier [96]. An ongoing clinical trial will determine whether KPT-8602 has equivalent therapeutic efficacy and/or better tolerability in cancer patients (NCT02649790).

## Conclusion and future directions

An important aspect of normal cell function, nuclear-cytoplasmic export, is often deregulated in cancers, providing a unique therapeutic opportunity. The recent FDA approval of selinexor for the treatment of RR multiple myeloma lends credence to this therapeutic strategy. Future expansion of the clinical indications of selinexor is contingent upon the success of additional clinical trials. More SINE compounds with improved efficacy and reduced adverse events will be tested and potentially approved for clinical use. In this light, extensive genetic and preclinical studies and well-designed clinical trials are the prerequisite to exploiting the full potential of XPO1 inhibitors in cancer treatment.

### Biological functions of XPO1

Identifying the MOA of XPO1 inhibitors is a constantly evolving process. Blockade of nuclear export of tumor suppressor proteins has been postulated as the major MOA. However, recent studies on the diversity and breadth of XPO1 cargoes outline a more complex view. Future endeavors to study the MOA rely on a comprehensive biochemical and genetic characterization of XPO1.

First, XPO1 is frequently overexpressed in human cancers, while XPO1 suppression has been shown to reduce the protein levels of driver oncogenes, such as MYC and EGFR, in multiple cancer types [97–100]. The detailed mechanisms of reciprocal regulation between XPO1 and driver oncogenes can be dissected using traditional genetic approaches [42]. Second, XPO1 is known to physically interact with hundreds of proteins, many of which may potentially influence its nuclear export activity [101]. Large-scale proteomic experiments coupled with loss-of-function genetic studies can illuminate the roles of the interacting proteins in regulating XPO1 nuclear export activity. Third, the recent deep proteomic study identifying > 1000 proteins exported by XPO1 [15] has significantly broadened our current perspective on XPO1 function. As RNAs have been increasingly recognized as the key XPO1 substrates, high-resolution transcriptomic studies should be carried out in a similar fashion to identify the multiple classes of RNA cargoes. This approach will provide a comprehensive view of XPO1 molecular function. Further comparison of cargoes associated with the wild-type and XPO1 mutant, such as XPO1^E571^, XPO^R749^, and XPO^D624^, will reveal novel gain of function in the mutants, potentially explaining their oncogenic activities, particularly in driving B cell malignancies. These genetic studies of XPO1 will lead to a better understanding of its role in tumorigenesis and the MOA of XPO1 inhibitors.

### Mechanisms of drug resistance

With FDA approval of selinexor for the treatment of multiple myeloma, there is an urgent need to investigate the mechanisms of drug resistance. Recurrent genomic mutations detected in patients’ tumors following treatment are associated with resistance. Investigating the causal roles of such mutations in drug resistance requires validation and further examination in cell culture and animal models. Additionally, the mechanisms of resistance can be more broadly studied through CRISPR-based genome-wide library screening [102]. CRISPR screens will identify a comprehensive list of candidate genes causally associated with drug resistance. Potential genetic or epigenetic mechanisms of resistance can be investigated in cell lines and animal models. The clinical relevance of identified candidates will be further examined in human tumor samples.

### Synergistic drug combinations

XPO1/CRM1 inhibitors are a unique class of drugs. Thus, their mechanisms of resistance may not be shared by other chemotherapeutic agents. This will provide vast opportunities to combine SINE compounds with other therapeutic modalities. Current clinical development of drug combinations typically relies on empirical testing, which only explores a small fraction of the potential combinations. High-throughput combinatorial screening using drug and/or genetic libraries, on the other hand, can examine millions of combinations in an unbiased manner [103]. As an example, small-molecule combination matrix screens can test XPO1 inhibitors in combination with hundreds of known drugs for systematic identification of synergistic, additive, and antagonistic interactions. Similarly, CRISPR genome-wide library screens can effectively identify potential drug synergies by simultaneous interrogation of thousands of drug targets [104]. Subsequently, therapeutic combinations discovered by such drug/genetic screens may be directly investigated in preclinical animal models and validated in clinical trials. While current preclinical and clinical studies have mainly focused on combining selinexor with existing standard treatments to ensure the timely translation of the findings to clinical applications, future efforts should focus on drug combinations with novel agents, including targeted and immunotherapeutic agents, to improve the efficacy and tolerability.

### Biomarkers of therapeutic responses and toxicity

Each human cancer type comprises a highly heterogeneous group of disease. Similar to what has been shown in targeted and immunotherapies, detailed tumor stratification based on genomic, pathohistological, and/or immunological characteristics can help delineate the specific subset of tumors responsive to XPO1 inhibitors. These biomarkers can be identified in preclinical genetic studies and clinical trials and subsequently used in the general patient population to prognosticate the subpopulations most likely to benefit from the therapy. In addition to the tumor-centric approach, pharmacogenomic studies of the patients on their therapeutic response and adverse events will pinpoint major genetic variants associated with inter-individual differences in drug metabolism and response [105, 106]. The availability of biomarkers predictive of therapeutic responses and toxicity will help identify the target patient population and exploit the full potential of XPO1 inhibitors in cancer treatment.

## Data Availability

Not applicable

## Abbreviations

AE: Adverse event
AML: Acute myeloid leukemia
CR: Complete remission
CRi: Complete remission with incomplete hematologic recovery
DLBCL: Diffuse large B cell lymphoma
MDS: Myelodysplastic syndrome
MOA: Mechanism of action
NES: Nuclear export signal
NHL: Non-Hodgkin lymphoma
OR: Objective responses
ORR: Overall response rate
PFS: Progression-free survival
RNP: Ribonucleoprotein
RR: Relapsed or refractory
SINE: Selective inhibitors of nuclear export
snRNA: Small nuclear RNA
